# Statistical Design of Overnight Trials for the Evaluation of the Number of Operating Rooms That Can Be Disinfected by an Ultraviolet Light Disinfection Robotic System

**DOI:** 10.7759/cureus.18861

**Published:** 2021-10-18

**Authors:** Bradlee Birchansky, Franklin Dexter, Richard H Epstein, Randy W Loftus

**Affiliations:** 1 Anesthesia, University of Iowa, Iowa City, USA; 2 Anesthesiology, University of Miami Miller School of Medicine, Miami, USA

**Keywords:** terminal cleaning, uv-c, hospital administration, operating room management, surgical site infections, ultraviolet disinfection, experimental design, monte-carlo simulation

## Abstract

Background and objective

The number of ultraviolet light disinfection robot systems that are needed for a facility’s surgical suite(s) and/or procedure suite(s) depends in part on how many rooms need to be disinfected overnight by each robot and how long this will take. The answer needs to be determined separately for each surgical and procedure suite because those variables vary both among facilities and among operating rooms or procedure rooms within facilities. In this study, we consider statistical designs to assess how many rooms a facility can reliably (≥90% chance) disinfect overnight using an ultraviolet light disinfection robot system.

Methods

We used 133,927 observed disinfection times from 700 rooms as a population from which repeated samples were drawn with replacement in Monte-Carlo simulations. We used eight-hour and 10-hour shift lengths being multiples of 40 hours for full-time hourly employees.

Results

One possible strategy that we examined was to estimate total disinfection times by estimating the mean for each room and then summing up the means. However, that did not correctly answer the question of how many rooms can reliably be available for the next day’s first case. Summing up a percentile (e.g., 90%) instead also was inaccurate, because the proper percentile depended on the number of rooms.

A suitable strategy is a brief trial (e.g., nine nights or 19 nights) with the endpoint being the daily number of rooms disinfected. Empirically, the smallest count of rooms disinfected among nine nights or the second smallest count among 19 nights are 10^th ^percentiles (i.e., ≈90% probability that at least that number of rooms can be disinfected in the future). The drawback is that while this approach gives the probability of a night with fewer rooms disinfected, it does not give information as to how many fewer rooms may either skip ultraviolet decontamination or start late the next workday because disinfection was not completed. Our simulations showed that there is a substantial probability (≥95%) of at most two rooms fewer or one room greater than the 10^th^ percentile with a nine-night trial and one room fewer or greater with a 19-night trial.

Conclusions

Because probability distributions of disinfection times are heterogeneous both among rooms and among treatments for the same room, each facility should plan to perform its own trial of nine nights or 19 nights. This will provide results that are within two rooms or one room of the correct answer in the long term. This information can be used when planning purchasing decisions, leasing, and technician staffing decisions.

## Introduction

Improving basic preventive measures in the perioperative field has been shown to substantially reduce *Staphylococcus aureus* transmission and surgical site infections [1]. These measures include better-quality environmental cleaning achieved in part by the targeted use of germicidal ultraviolet light disinfection [1]. More work is needed to facilitate the adoption of this enhanced cleaning approach because disinfection times vary substantively among operating rooms and among cycles within rooms [2].

The decision as to how many ultraviolet light disinfection robot systems are needed for a facility’s surgical suite(s) and/or procedure suite(s) depends in part on how many rooms need to be disinfected overnight by each robot and how long this will take. The answer needs to be determined separately for each surgical suite and procedure suite because those variables vary both among facilities and among operating rooms or procedure rooms at facilities [2]. The answer is important because buying too many is wasteful and buying too few will not allow the necessary number of rooms to be disinfected nightly. Also, answering the question with the fewest trials is important because the devices differ in features, and trials may be performed with a loaned device.

Suppose that the times for an ultraviolet system to disinfect individual operating rooms at a facility’s surgical suite were essentially constants (e.g., room A: 15 minutes and room B: 25 minutes). Additionally, suppose that the time to wheel the robot to an adjacent operating room, and press buttons on its tablet, was also a constant (e.g., 10 minutes). Each room’s constant time would not be known a priori; rather, one would learn that room A takes 15 minutes and room B takes 25 minutes through actual use. Therefore, a trial would still be needed to figure out how many robotic systems are needed to disinfect all operating rooms overnight (e.g., 9:00 PM to 6:00 AM including two half-hour breaks). However, if the time were a constant, the trial could be just one night long. A one-night trial would be sufficient because the result would apply to all other nights. However, the reality is that the times to clean specified rooms are not constant, but rather are random variables, differing not only among rooms but substantially among disinfections for the same room (e.g., from the heterogeneity of equipment in the room) [2]. Thus, more than one day of observation is necessary.

The aim of the current study was to evaluate statistical designs of trials to assess how many rooms that a facility can disinfect reliably overnight (≥90% chance) [3]. It is desirable to choose a trial length that is not excessively long, but not too brief that it produces an unreliable result. The design is not that of a clinical trial to be performed once, but rather of trials to be performed separately for each surgical suite or procedure suite (e.g., endoscopy center). For example, consider a trial of nine nights with a robotic system delivered on a Monday and used through the following Friday morning. Among those nine nights, record the numbers of rooms that the facility can disinfect nightly. Take the minimum of the nine observations (i.e., rooms disinfected). There is a ≈90% probability that the future rooms that the facility can disinfect nightly would exceed that minimum [4-8]. Our goal was to evaluate the precision of the estimate of the 10^th^ percentile of rooms disinfected when obtained with just nine nights or 19 nights of data; the rationale for these choices is described in the Materials and methods section. The results of our study can be used by responsible operating room and facility managers, companies selling or leasing the products, and financial engineers responsible for the agreements and for choosing whether to plan eight-hour or 10-hour night shifts when using ultraviolet light disinfection devices for terminal cleaning [3].

## Materials and methods

The University of Iowa Institutional Review Board informed on May 20, 2021, that this project does not meet the regulatory definition of human subjects research. We prepared the paper and this methods section following the Strengthening the Reporting of Empirical Simulation Studies (STRESS) guidelines for simulation [9].

Data used for the simulations

The data used were the same as for our recent study on how to predict the times for disinfection of single rooms (e.g., one operating room in the middle of the workday) [2]. The studied ultraviolet light disinfection system (Helios®, Surfacide, Waukesha, WI) consisted of one or more towers that were connected by Bluetooth wireless technology to a tablet computer running the Android operating system (Google, Mountain View, CA). We studied three towers as recommended for rooms with a single bed (e.g., operating rooms). Each tower is a 2-meter-tall mechanical robot working vertically and circumferentially. Details are provided in the Appendix. We used the 133,927 observed disinfection times as a population from which repeated samples were drawn with replacement [2].

Computer simulation in MATLAB® R2020a (The MathWorks, Inc., Natick, MA) was used for three objectives, each listed in the following three sections: to compare the 90% upper prediction limits for disinfection time of multiples of rooms to the mean disinfection times; to determine how many rooms can be reliably disinfected in ≤8 hours and ≤10 hours; and (our primary objective) to determine the precision of trials of different numbers of nights to show how many rooms can be reliably disinfected. The primary objective is described third because the first two objectives determined which trial designs were simulated, as well as the methodology we used for the trial simulations.

Does the number of rooms that can reliably (90%) be disinfected overnight differ from the average number of rooms disinfected overnight?

**Figure 1:**
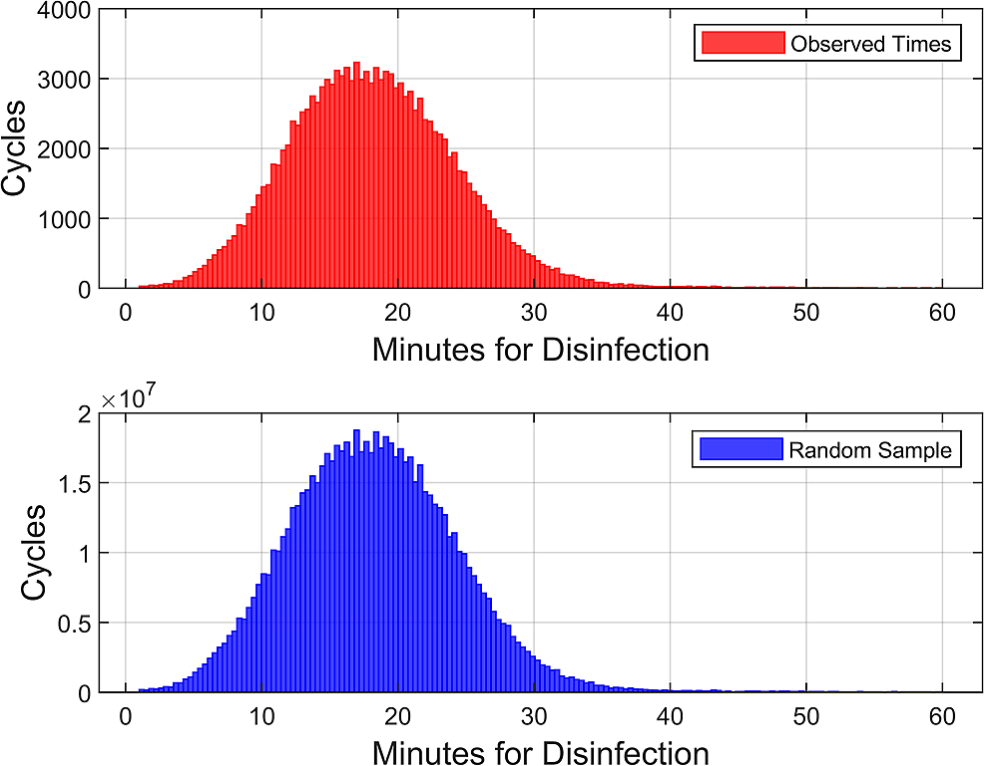
Probability distribution of minutes for disinfection The figure shows the observed times for disinfection (top) and simulated times for disinfection (bottom). Regarding the observed disinfection times, the mean was 18.3 minutes. The 90^th^ percentile was 26 minutes. The probability distribution is seen to be unimodal, with moderate right skewness (0.904). The 96/133,927 observations (0.07%) longer than one hour (range: 61-72 minutes) were excluded from the figure, as their frequency was exceedingly small. To create the histogram for simulated times for disinfection in this figure, we showed only the first 779,999,740 values, where 779,999,740 = (3 × 10^6^ - 1) simulations × 26 rooms × 10 replications

The raw data used consisted of one column having the disinfection times and another column with the matching number of observations of treatments in the room, stored in a 133,927 × 2 matrix. For example, if room 1 had 50 observations, then for 50 of the 133,927 rows, all 50 entries in the second column were of value 50. Pseudo-random samples of disinfection times were taken with replacement using the MATLAB® datasample function, with each observation weighted inversely to its room’s total number of treatments (i.e., frequency). Thus, all rooms were weighted equally. For example, if room 1 had 50 observations and Room 2 had 100 observations, each observation in room 1 was assigned a weight of 1/50, and each observation in room 2 was assigned a weight of 1/100. To ensure reproducibility of results, the pseudo-random number generator seed was initialized before the first simulation. The rooms were weighted equally, not any two disinfection times, because simulations were of the times to disinfect multiple rooms with one disinfection per room, and because disinfection times were correlated within rooms. Figure 1 makes the comparison between observed and simulated times.

We defined two terms for use henceforth: replication and simulation. In each replication, a pseudo-random sample of (3 × 10^6^ - 1) × 26 disinfection times was drawn from the population with replacement, per the preceding paragraph. The replications were used to generate a sampling distribution of the calculated statistics (i.e., means, 90^th^ percentiles, and 94.87^th^ percentiles, see below). The sampling distributions assessed whether the numbers of simulations (i.e., N = 3 × 10^6^ - 1) were sufficient for the standard deviations of these statistics to be less than one-fourth of each reported smallest digit. One simulation referred to the summing up of disinfection times of one multiple of rooms, the multiple ranging in size from one to 26 rooms. For example, when the number of rooms being simulated was five rooms, each simulation was a sum of the disinfection times of five rooms. (The time to disinfect a given room was treated as independent of the time to disinfect another.) Thus, with 3 × 10^6^ - 1 simulations, the first 3 × 10^6^ - 1 multiples of five disinfection times out of the (3 × 10^6^ - 1) × 26 total disinfection times in the pseudo-random sample were summed up and stored. When the number of rooms being simulated was 26 rooms, each simulation was a sum of the disinfection times of the 26 rooms. The result was 26 arrays (i.e., one for each number of rooms, 1-26), each of length 3 × 10^6^ - 1 (i.e., the number of simulations), with each value in the array a sum of disinfection times. That is, the first array had sums of disinfection times for one room, the second contained sums of disinfection times for two rooms, and so forth. Means and 90^th^ and ((1-0.1)^(1/2)^) ≈ 94.87^th^ upper prediction limits were computed for each of the 26 arrays of 3 × 10^6^ - 1 values. Applying Šidák’s correction, the 94.87^th^ percentile gave the probability that two independent sets of devices would each complete, with 90% probability, disinfection of a given number of rooms [10]. The reported statistics (i.e., means, 90^th^ percentiles, and 94.87th percentiles) are means among the 50 replications. We limited all reported values to those with standard deviations that were less than one-quarter of the smallest reported decimal.

Prediction limits were calculated using the formula *p* × (*N*+1) = *p* × (3 × 10^6^ - 1 + 1), where *p* is the proportion (e.g., 0.90) and *N* is the number of simulations [4-8,11]. *N* = 3 × 10^6^ ‑ 1 simulations were used because *N* + 1 is a multiple of 10, and thus the 90^th^ percentile is precisely the 2.7^th^ million value, with no interpolation required. Thus, among the 3 × 10^6^ - 1 simulations, the “90^th^ percentile” means the 2.7 × 10^6^ value and the “94.87^th^” percentile means the 2,846,050^th^ value.

Inter-room “setup” times were included in the calculations. That is, one room’s disinfection cannot end and the next start at once. The towers must be moved from room to room, and the next disinfection cycle started. In these calculations, we considered this process of unplugging the towers, joining them into a set, pushing them to the next room, positioning them, plugging them in, and then clicking on the tablet to take 10 minutes. Thus, for two rooms, we included a fixed 10 minutes of setup time, for three rooms 20 minutes of setup time, and so forth. The assigned technician prepares the next room for the towers (e.g., small movements of equipment in the room obscuring access to convenient electrical outlets) during the disinfection cycle of the current room. The technician may also return to the preceding room for any equipment adjustments. (Note: the choice of the setup time does not influence the results of our primary objective, see below).

We simulated a maximum of 26 rooms because we were interested in the number of rooms that can reliably be disinfected in one night. Based on the mean disinfection time for one room being 18.3 minutes (Figure 1) and a reasonable inter-room transition and setup time of 10 minutes, 26 rooms × 18.3 minutes/room + 25 movements of robot × 10 minutes per move = 12.1 hours, a maximum nighttime shift duration.

The run time for completion of the 50 replications was less than three hours on the University of Iowa’s virtual desktop.

Does the definition of reliable (e.g., 90% or 75%) affect how many rooms can be disinfected in eight hours or 10 hours?

This second question was answered by using the same simulation process as the first. Again, there were 26 arrays (i.e., one for each number of rooms, one to 26), each of length 3 × 10^6^ - 1 (i.e., the number of simulations), with each value in the array a sum of disinfection times. That is, the first of 26 arrays held sums of disinfection times for one room, the second contained sums of disinfection times for two rooms, and so forth. The endpoints used were the percentage of simulations with total disinfection time ≤8 hours and the percentage of simulations with total disinfection time ≤10 hours. We calculated the mean of 50 replications of the observed percentages of simulations for each multiple of rooms with total disinfection time ≤8 hours and the percentage of simulations for each multiple of rooms with total disinfection time ≤10 hours. Among the one to 26 rooms, the maximum standard deviation among replications of the fraction completed in ≤8 hours and ≤10 hours was less than 0.030%.

Primary study objective: a statistical design for a facility to estimate how many of its rooms can reliably be disinfected

Our primary study objective was to determine the suitability of trials of length nine nights or 19 nights, compared with a long period (e.g., 249 nights), for a facility to reach the correct answer to how many of its rooms can reliably be disinfected in an eight-hour or 10-hour nighttime shift. The data used for the simulations were the same. Trials need to be used because each facility has a finite number of rooms and its own probability distribution of inter-room setup times (i.e., facilities cannot directly use the results of the preceding two sections).

We were interested in disinfection during nights because that is when most operating rooms are unused. We used shift lengths that were multiples of 40 hours for full-time hourly employees. We did not consider longer, 12‑hour shifts because, after disinfection, the rooms need to be set up for the first cases of the day. A period such as 6:00 PM to 6:00 AM would be unrealistically early for terminal cleaning at many hospitals.

We simulated 26 rooms and recorded the number of rooms that had disinfection completed in ≤8 hours and ≤10 hours. For the nine-night trials, each simulation of 26 rooms was performed another eight times, making a total of nine nights. For the 19-night trials, each simulation was performed another 18 times. For the 249-night trials, each was performed another 248 times. The resulting nine, 19, and 249 values were each sorted in ascending order. The 10^th^ percentiles (i.e., 1^st^, 2^nd^, and 25^th^ smallest values for the nine-, 19-, and 249-night trials, respectively) were stored. This process was replicated 99,999 times, giving a total of 100,000 replications. Because 1 = 0.1 x (9+1), 2 = 0.1 x (19 + 1), and 25 = 0.1 x (249 + 1), precisely, with no rounding, the 10^th^ percentiles could be estimated from the trials without error [5]. The choice of 26 rooms was a suitable maximum number of rooms to simulate because it was not found for any of the 100,000 replications that 26 rooms were disinfected within 10 hours. The results were presented as histograms of the 100,000 replications of the 10^th^ percentiles. Using the 100,000 replications, asymptotic standard errors of the histograms’ proportions were calculated [5].

Fragility analysis was used to evaluate the choice of 100,000 replications. All 10^th^ percentiles (i.e., numbers of rooms, such as 13, 14, or 15) differed by more than 500 replications from a threshold of the 2.5^th^ or 97.5^th^ percentiles of the 100,000 generated observations. Thus, the numbers of rooms disinfected per replication would have needed to differ in more than 500 replications to change histogram widths.

Sensitivity analyses were then performed, repeating the simulations while using inter-room setup times of 7.5 minutes and of 12.5 minutes.

The run time for completion of 100,000 replications for each of the nine-night, 19-night, and 249-night simulations was approximately 20 minutes in total.

## Results

Numbers of rooms that can reliably (90%) be disinfected overnight differs from the average number of rooms that can be disinfected overnight

**Figure 2:**
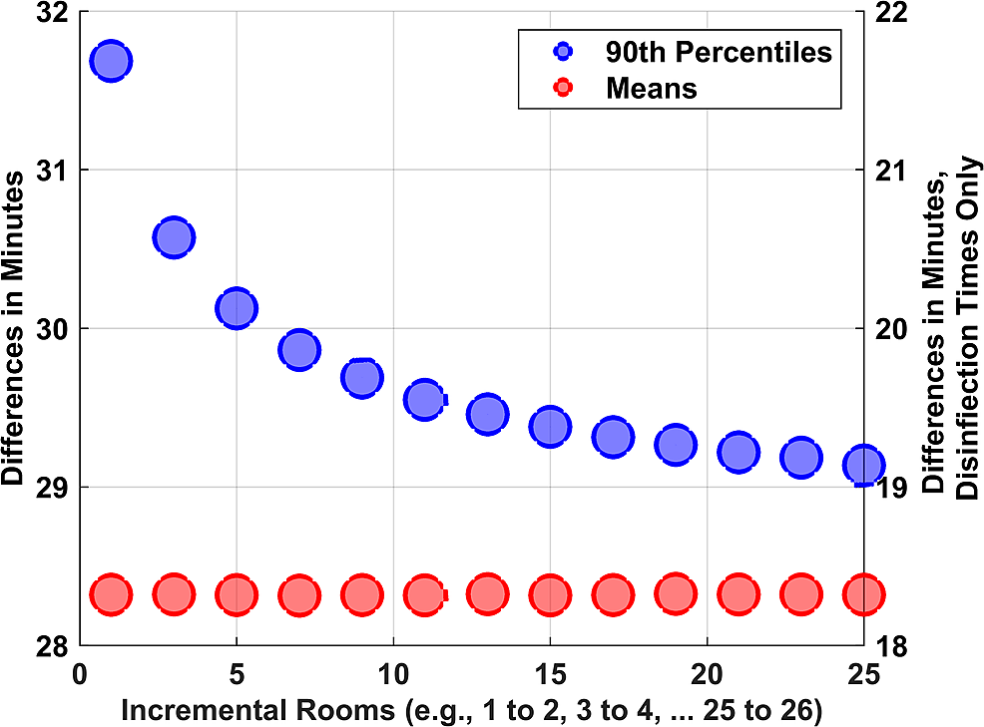
Additional time to reliably disinfect each extra room decreases with greater numbers of rooms Numerical values are given in the paper's table. Blue dots show the pairwise differences between 90^th^ percentiles. Red dots show the differences between means. The left-sided vertical axis includes setup times. The right-sided axis does not. The differences of 90^th^ percentiles (blue) all being greater than the differences of means (red) is important. The two curves have not merged by the numbers of rooms that can be disinfected in eight hours or 10 hours. The consequence is that a facility cannot use the mean disinfection time of one room as an accurate estimate of the incremental time that will be required to reliably disinfect an additional room. The plotted data are the means of 50 replications. Error bars were excluded because the standard deviations among replications were less than 0.0413 minutes for all displayed values. In other words, all reported values contain their 95% confidence intervals

The mean times to disinfect multiple rooms were nearly additive, shown by the red markers in Figure 2, being nearly perfectly flat [12,13]. The range of differences of means among replications was ±0.01 minutes. Thus, for example, with the average time to disinfect one room being 18.3 minutes, the average for 13 rooms was 237.9 minutes, where 237.9 minutes = 13 × 18.3 minutes. These results show that the simulations have face (content) validity.

**Figure 3:**
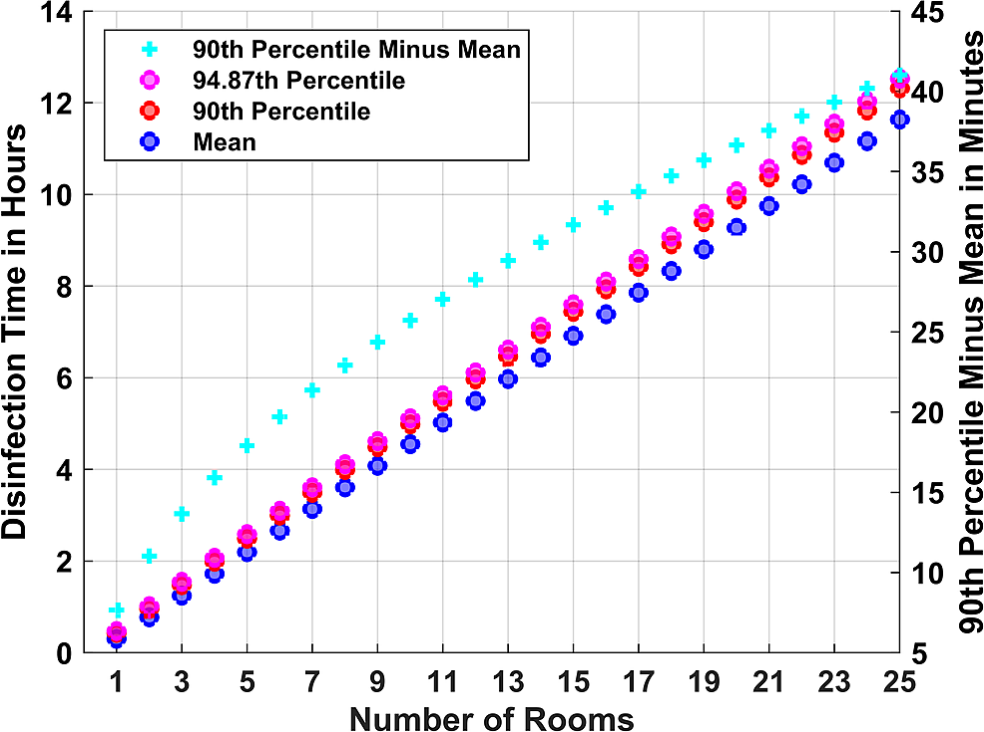
Mean cannot be used to reliably predict disinfection time for a number of rooms The computer simulations show that the 90^th^ percentile of the time for disinfection increased approximately linearly with the numbers of rooms, just as did the mean. However, the cyan crosses symbols show that the relationship between the 90^th^ percentile and the mean was not that the 90^th^ percentile was the mean plus a constant safety factor. The purple circles are 94.87^th^ percentiles (i.e., 90% upper prediction limits for the time that would be needed for each of two independent sets of three towers to reliably disinfect the given number of rooms) [10]. Error bars were excluded because the standard deviations among 50 replications were less than 0.06 minutes for all displayed values. In other words, all reported values contain their 95% confidence intervals. All values shown include fixed, 10-minute, setup times

Many facilities will have a preset number of rooms to be disinfected (e.g., the daily numbers of operating rooms staffed). The statistical endpoint of interest is the time to plan to disinfect those rooms. What is necessary is to provide a sufficient interval so that those multiple rooms can be disinfected reliably (e.g., 90%) [8,13,14]. Figure 2 shows that the 90^th^ percentile for the time to disinfect one room was 26.0 minutes. (There were sufficient simulations for the standard deviation among replications to be less than 0.01 min). Figure 2 also shows that the incremental times for each extra room to be disinfected were progressively less when keeping a 90% chance of completing the added room. However, from the far right of Figure 2, the incremental times for the 90^th^ percentiles do not approach those for means by the number of rooms that can reliably be disinfected in eight hours or 10 hours. Table 1 presents the values from Figures 2, 3 in a readable format.

**Table 1:** Values from Figures 2, 3 in a readable format, with times reported in units of minutes ^a^Rooms to be disinfected with three towers (i.e., one unit), based on 10 minutes for moving the towers and then using the tablet to start their cycle (i.e., “setup” time). The table can be adjusted for any other setup time. For example, if a facility has 15.5-minute setup times rather than 10 minutes, then add 5.5 minutes x (number of rooms - 1) to all listed values ^b^The differences in the 90^th^ percentiles between rows match those shown in Figure 2. All time values in all columns are rounded to the nearest integer ^c^The 94.87^th^ percentiles are the 90% upper prediction limits for the time that would be needed for each of two independent sets of three towers to reliably disinfect the given number of rooms [10]. The standard deviations among 50 replications were less than 0.06 minutes for all listed values in all columns. In other words, 95% confidence intervals would all be at most ± 0.12 minutes

Number of rooms^a^		Mean (minutes)		90^th^ percentile (minutes)^b^		94.87^th^ percentile (minutes)^c^
1		18		26		28
2		47		58		61
3		75		89		93
4		103		119		124
5		132		150		155
6		160		180		186
7		188		210		216
8		217		239		247
9		245		269		277
10		273		299		307
11		302		329		337
12		330		358		367
13		358		388		397
14		386		417		426
15		415		446		456
16		443		476		486
17		471		505		515
18		500		535		545
19		528		564		575
20		556		593		604
21		585		622		634
22		613		652		663
23		641		681		692
24		670		710		722
25		698		739		751

For example, consider a technician with 13 rooms to disinfect. Using a setup time of 10 minutes, at least 388 minutes would be needed to have a 90% chance of an on-time completion (Figure 3). To complete disinfection of the 13 rooms before 7:00 AM, with one ultraviolet light disinfection robot of three towers, disinfection of the first room would have to start by 12:32 AM, where 12:32 AM = 7:00 AM - 388 minutes. The 90^th^ percentile, 388 minutes, was longer than 357.9 minutes, the sum of 13 times the mean of 18.3 minutes plus 12 turnover (setup) times of 10 minutes each. However, 388 minutes was also less than 458.0 minutes, the sum of 13 times the 90^th^ percentile for one room plus 12 setups times of 10 minutes each. Thus, Figure 2 shows that a facility cannot accurately use any single choice for the time to disinfect (e.g., mean or 90^th^ percentile) and then apply that constant for multiple rooms [13]. This explains why we subsequently considered, instead, the use of eight-hour or 10-hour long trials, see below.

Definition of reliable (e.g., 90% or 75%) does not affect how many rooms can be disinfected in eight hours or 10 hours

**Figure 4:**
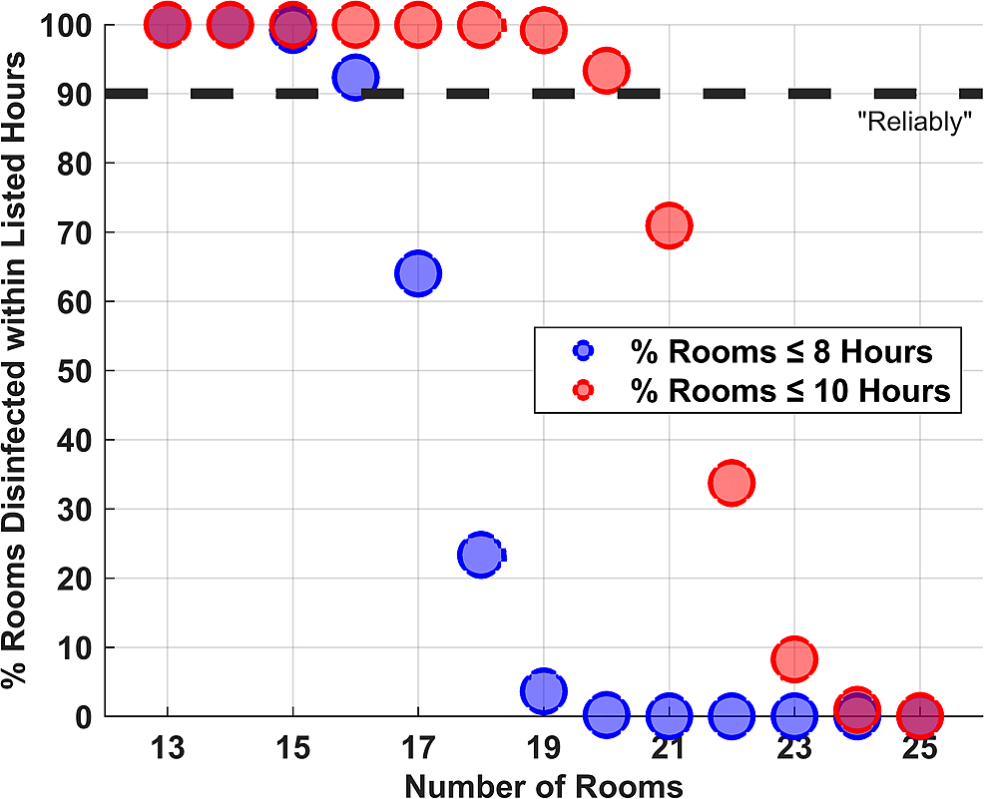
Ultraviolet light disinfection robot can reliably disinfect 16 and 20 rooms in eight hours and 10 hours, respectively This figure shows that on more than 90% of days, 16 rooms can be disinfected in eight hours and 20 rooms can be disinfected in 10 hours. Knowing these numbers of rooms, 16 and 20, is important for the interpretation of the study’s primary result. From the current figure, error bars were excluded because the standard deviations among the 50 replications were less than 0.030% for all displayed values. In other words, all reported values contain their 95% confidence intervals. All values include fixed 10-minute setup times

With 10-minute setup times, 16 rooms could be disinfected reliably (90^th^ percentile) in eight hours and 20 rooms in 10 hours (Figures 3, 4). Thus, facilities using the ultraviolet disinfection robot with three towers and with more than 16 or 20 rooms to be disinfected during an eight-hour or 10-hour night shift would need more than one robot (e.g., six towers). Applying Šidák’s correction, the 94.87^th^ percentile (Figure 3) gives the probability that two independent sets of devices would each complete, with 90% probability, a given number of rooms [10].

Answering the question used for the primary study objective, below, the number of rooms that could be disinfected reliably in eight hours or 10 hours was insensitive to the choice of “reliable,” 90%, 80%, or 75% probability of completion. The reason was that the descent in the probability of completion within eight hours or 10 hours was precipitous after 16 or 20 rooms, respectively (Figure 4). Thus, regardless of the choice of 90%, 80%, or 75% as reliable, the correct answer was that 16 rooms could be reliably disinfected in ≤8 hours, and 20 rooms in ≤10 hours. Differences in setup times among facilities (e.g., longer if rooms to be disinfected are not adjacent) would change how many rooms can be disinfected, but not the shapes of the curves in Figure 4. Thus, our results show that over an extended period of data collection (e.g., one year), little statistical uncertainty is expected as to how many rooms can be reliably disinfected at each facility during a night shift.

Primary study objective: a statistical design for a facility to estimate how many of its rooms can reliably be disinfected

Our primary objective was to show how each facility can estimate from its own data how many rooms the technicians can reliably disinfect with each ultraviolet disinfection robot [3]. For example, technicians may have non-adjacent rooms or frequent interruptions. The corresponding aim for a company with disinfection systems is to have the briefest possible but accurate trial duration to learn how many robots are needed at a facility (e.g., to provide disinfection as a service). While the specific results can differ from our Figures 2-4 (e.g., different setup times from geography), our primary goal is the experimental design (i.e., not affected by the inter-room “setup” times).

**Figure 5:**
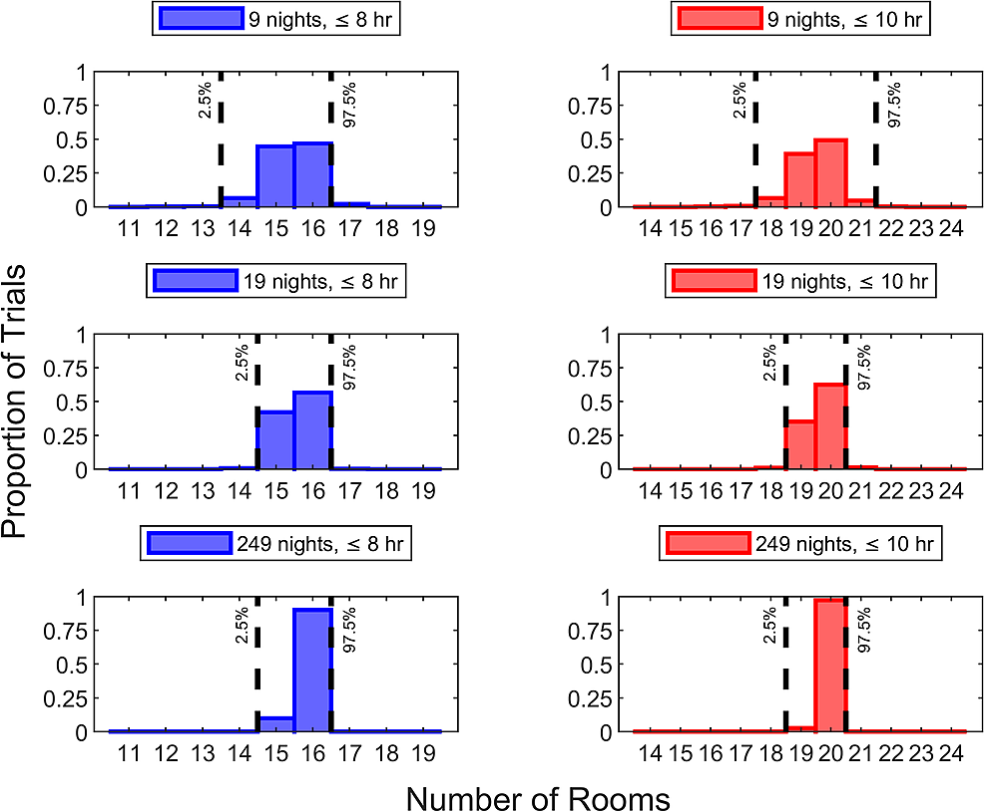
Proportion of simulated trials for which the plotted numbers of rooms disinfected were the 10th percentile among days in the trial The number of rooms disinfected in ≤8 and ≤10 hours on each of the 100,000 simulated nine-night, 19-night, or 249-night trials were sorted. For nine nights, the histograms show the 100,000 smallest numbers of rooms disinfected. For 19 nights, the second smallest numbers of rooms are shown. For 249 nights, the 25^th^ smallest numbers of rooms disinfected are shown. Regarding the vertical axis, each box of the histogram lacks an error bar because they would all be imperceptibly small, each having a standard error of less than 0.0016 (proportion). This figure uses an inter-room setup time of 10 minutes, as compared with 7.5 minutes in Figure 6 and 12.5 minutes in Figure 7

We considered nine nights as the minimum trial length because the 90^th^ percentile obtained by a facility would precisely equal the minimum of the observed rooms disinfected during nights [i.e., (1-0.90) x (9+1) = 1]. For example, an ultraviolet disinfection robot system is delivered on a Monday morning and collected nine days later, on Friday morning. Simulations showed that using a nine-night trial (i.e., two work weeks), a facility can empirically arrive at the correct answer (e.g., 16 rooms for ≤8 hours and 20 rooms for ≤10 hours, from Figure 4), one or two rooms less than the correct answer, or one room greater than the correct answer with >95% probability, specifically ≥99% (Figure 5). With a 19-night trial (i.e., four work weeks), the probability of determining the correct numbers of rooms, one less, or one more was >95%, specifically ≥99% (Figure 5).

**Figure 6:**
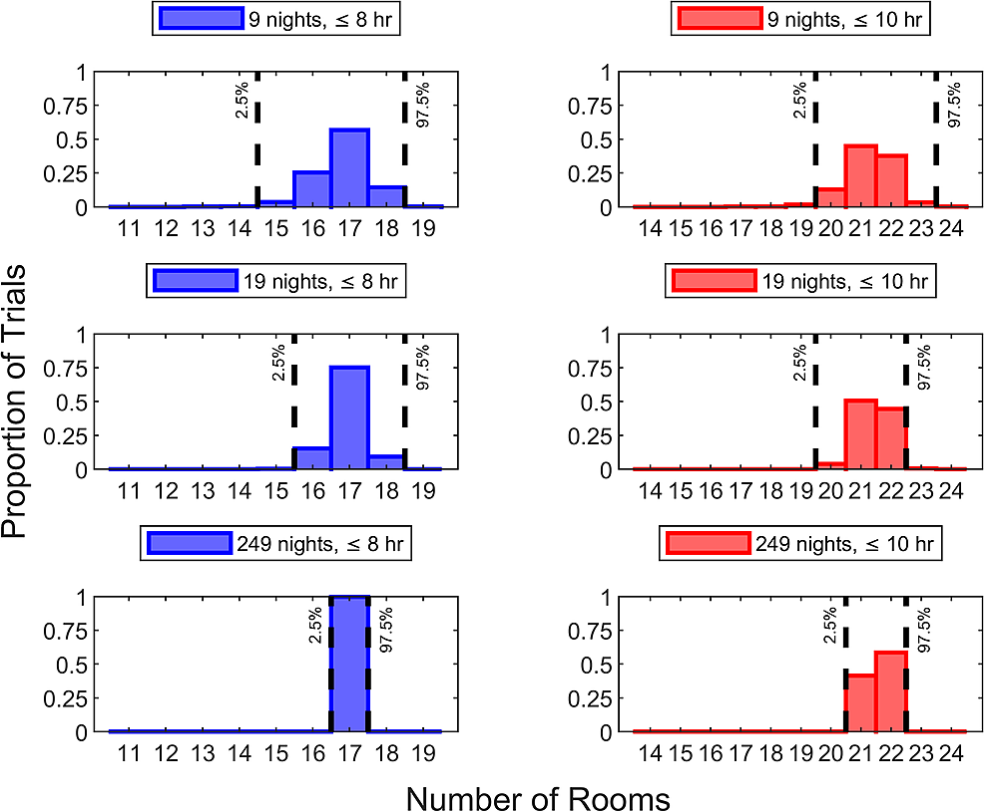
Sensitivity analysis repeating Figure 5 but using inter-room setup time of 7.5 minutes instead of 10 minutes Repeating Figure 4 to determine how many rooms can reliably (>90%) be disinfected using a three-tower ultraviolet light robotic disinfection system within eight hours, it was 17 rooms for 96% of nights and 18 rooms for 81% of nights (i.e., correct answer of 17 rooms). For >95% of nine-night trials, the 10^th^ percentile (i.e., the smallest number of rooms among the nine nights) was between two rooms less and one room more than 17 rooms. Similarly, for >95% of 19-night trials, the 10^th^ percentile (i.e., the second smallest number of rooms among the 19 nights) was between one room less and one room more than 17 rooms. Repeating Figure 4 for 10 hours, there could be disinfection of 21 rooms for 98% of nights, 22 rooms for 91%, and 23 rooms for 68%. For >95% of nine-night trials, the 10^th^ percentile was between two rooms less and one room more than 22 rooms. For 96% of 19-night trials, the 10^th^ percentile was 22 rooms, one fewer, or one more

**Figure 7:**
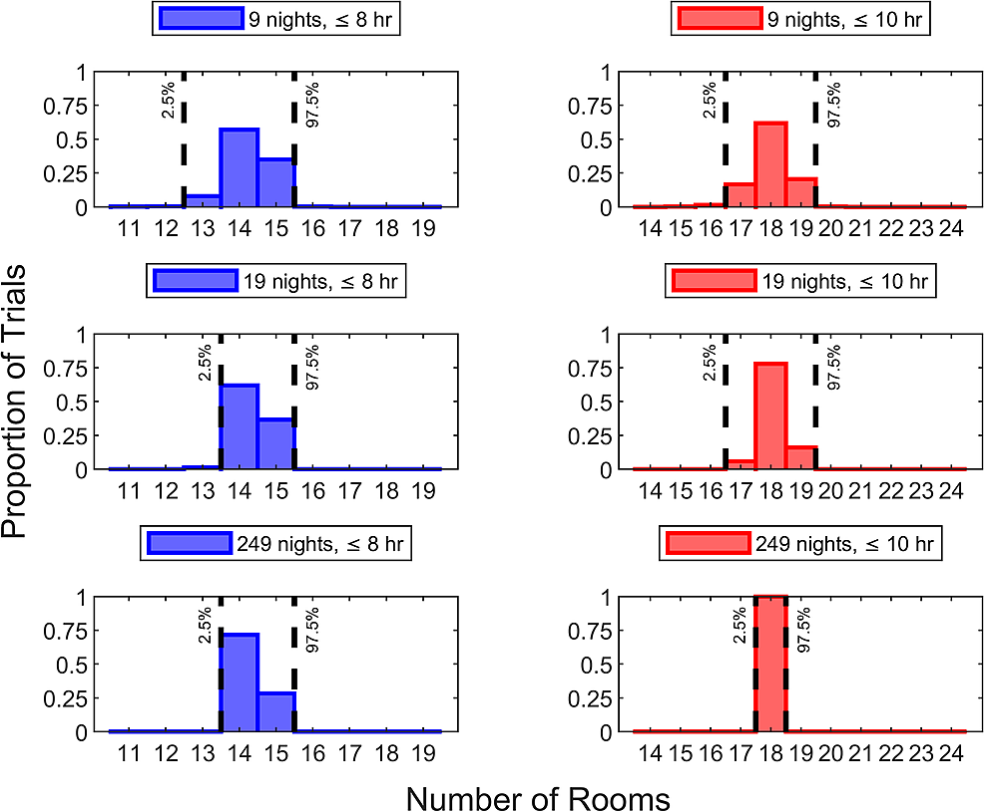
Sensitivity analysis repeating Figure 5 but using inter-room setup time of 12.5 minutes instead of 10 minutes Repeating Figure 4 to determine how many rooms can reliably (>90%) be disinfected using a three-tower ultraviolet light robotic disinfection system within eight hours, it was 14 rooms for 99% of nights and 15 rooms for 89% of nights (i.e., correct answer of 14 rooms). For >95% of nine-night trials, the 10^th^ percentile (i.e., the smallest number of rooms among the nine nights) was between two rooms less and one room more than 14 rooms. Similarly, for >95% of 19-night trials, the 10^th^ percentile (i.e., the second smallest number of rooms among the 19 nights) was between one room less and one room more than 14 rooms. Repeating Figure 4 for 10 hours, there could be disinfection of 18 rooms for 98% of nights and 84% for 19 rooms. For >95% of nine-night trials, the 10^th^ percentile was between two rooms less and one room more than 18 rooms

Inter-room setup times may differ among facilities. Both with briefer (Figure 6) and longer (Figure 7) inter-room setup times, conclusions were the same (i.e., >95% probability of being within two rooms of the correct answer with a two-week trial and one room with a four-week trial).

## Discussion

Ultraviolet disinfection treatment can be used as part of a multifaceted approach to reduce surgical site infections, with operating rooms disinfected following known pathogen exposure [1]. The strategy can potentially be extended to severe acute respiratory syndrome coronavirus 2 (SARS-CoV-2) [15-17]. We have previously described how to quantitatively schedule such individual uses of ultraviolet disinfection robotic systems [2]. In the current paper, we considered the different problem of using ultraviolet disinfection robots for terminal cleaning of multiple adjacent rooms [18].

To keep the productivity of first-case starts [19], facility managers need to focus on the accuracy of finishing disinfection treatments by the scheduled start time of mornings’ surgical cases. We showed previously that the accuracy of predictions cannot be realized for a facility’s surgical suite, hospital ward, etc., by using averages from other facilities (e.g., estimated times from the vendor) [2]. The reason is that disinfection times vary markedly among rooms and among treatments of the same room (e.g., from heterogeneity in the equipment in the room). Consequently, treatment duration data are needed for the individual rooms of interest [2]. None of the standard probability distributions applied (e.g., normal, log-normal, gamma, Weibull), precluding the use of Bayesian strategies used in other operating room management problems [2,8].

One possible strategy that we examined in the current study was to estimate total disinfection times by estimating the mean for each room and then summing up the means (Figure 3). However, that does not correctly answer the question of how many rooms can reliably be disinfected nightly (i.e., so that the room is available the next morning) (Figure 2). Summing up instead a percentile (e.g., 90%) also would be inaccurate, because the proper percentile depends on the numbers of rooms (Figure 2). If a facility is choosing how many robotic systems to buy, or lease, and it already has extensive prior data for each room, then a computer simulation study like we did could be used (Figure 4). For facilities without prior use of the disinfection robotic system, we considered, instead, brief (e.g., nine nights or 19 nights) trials with the endpoint being the daily numbers of rooms disinfected. Empirically, the smallest count of rooms disinfected among nine nights or the second smallest count among 19 nights are guaranteed to be 10^th^ percentiles (i.e., 90% probability that at least that number of rooms can be disinfected in the future). The drawback is that while this approach gives the probability of a night with fewer rooms disinfected, it does not give information as to how many fewer rooms may either skip ultraviolet decontamination or start late the next workday because disinfection was not completed (Figure 4). Our results show that there is a substantial probability (≥95%) of at most two rooms fewer or one room greater than the 10^th^ percentile with a nine-night trial and one room fewer or greater with a 19-night trial (Figures 5-7). This information can be used when planning the purchasing decision, leasing decision, or technician staffing decision [3].

Limitations

Like many Monte-Carlo simulation studies for designing clinical or engineering trials, our work was limited by the knowledge of the probability distributions. We assumed from prior empirical results that we could select randomly from among the 700 rooms of disinfection times available [2]. We lacked data on setup times for physically adjacent operating rooms [20] and instead relied on our personal experience. However, our results should in no way be interpreted as providing the answer to how many operating rooms or procedure rooms that a facility can disinfect nightly; see heterogeneity of counts of rooms among Figures 5-7. Even under ideal conditions, it is seemingly implausible to estimate how many rooms can reliably be disinfected by using a single summary statistic for disinfection time and summing up among rooms or alternatively estimating the probability distribution for each room and then simulating (Figures 2-4). Instead, a brief trial of at least nine nights should be performed at the facility. Validity of that conclusion can be expected because of their close analogy to findings for other similar duration tasks. When the same approach is used for estimating lower prediction limits of operating room case durations, there too is a substantial benefit of increasing from a sample size of nine to 19, because then the second smallest (or largest depending on context) can be used instead of the smallest (or largest) [8].

Our study was limited to the question of how many rooms can be disinfected, not which rooms should be disinfected. The rooms with the most surgical site infections per month should be selected [21]. Those operating rooms can be strikingly different from the rooms with the largest incidences of surgical site infection [22]. The reasons are the non-random distributions among operating rooms of cases’ surgical specialties, case durations, case urgency, and patient physical status [23]. Thoughtful selection of rooms to disinfect is warranted because, from an observational study, there are orders of magnitude of inequality in the numbers of surgical site infections per month among operating rooms and specialty combinations [22]. Once a hospital has screened its electronic medical record data to know the operating rooms with the most surgical site infections, a suitable next step is to measure the incidence of *Staphylococcus aureus* transmission both among sites within the operating room during the same surgical case and then among successive cases in the room [21,24]. Swab and culture the specific locations in the operating room and at specific times (e.g., the adjustable pressure valve of the anesthesia machine at the end of the surgical case) to quantify environmental contamination [25]. Ultraviolet disinfection should include those operating rooms with *Staphylococcus aureus* transmission [1].

## Conclusions

We evaluated how a facility can determine how many of its rooms can be disinfected reliably in an eight-hour or 10-hour period by an assigned technician. We showed that this is not the same question as figuring out how many rooms can be disinfected on average. The choice of determining the 10^th^ percentile of the number that can be disinfected nightly is appropriate, with a lack of sensitivity to the precise proportion. Because probability distributions of disinfection times are heterogeneous both among rooms and among treatments for the same room, using a nine-night or 19-night trial is a rational approach. Expect that on the 10% of future days when a nine-night trial’s minimum is an underestimate, the underestimation would be by one or two rooms, and if an overestimate by one room. Using a 19-night trial, on the 10% of future days when the second smallest count of rooms is an underestimate, expect that to be by one room, and if an overestimate also by one room.

## Appendices

Data used for the simulations, details

The studied ultraviolet light disinfection system (Helios®, Surfacide, Waukesha, WI) consisted of one or more towers that were connected by Bluetooth wireless technology to a tablet computer running the Android operating system. When the tablet was connected to the towers, every treatment performed since the last upload was added to the Surfacide database. Therefore, the data had no missing values. The towers did not run without the tablet and the tablets required connection to the Surfacide internet portal at least every 30 days to remain operational.

The treatment durations were those uploaded from the earliest date (January 1, 2015) through May 6, 2021 [2]. From among the 2,058,095 records, we excluded 776,397 treatments done with fewer or more than three towers, 629,123 treatments without a calculated duration completed (e.g., the user chose to override the estimated duration or paused/aborted the treatment), 102,441 that used higher than normal energy level (e.g., for Clostridium difficile spores), 19,758 with scrub mode to add time for greater treatment of individual pieces of equipment in the room, 430 with “bathroom only” mode, and 62 with implausible date/times caused by failure to reset the tablet’s system clock if the battery drains fully [2]. Among those 529,884 disinfections, there were 700 rooms each with N ≥100 treatments, from 68 organizations and totaling 133,927 disinfection times [2]. The times were integers in units of seconds. In the previous study, we showed that there was no association between rooms’ mean disinfection times and numbers of treatments (Kendall’s 𝛕_b_ = -0.02, P = 0.48), means by organization and their total treatments (𝛕_b_ = 0.04, P = 0.61), rooms (𝛕_b_ = 0.07, P = 0.43), or disinfection time (𝛕_b_ = 0.13, P = 0.11) [2].
